# Effects of urbanization on productivity of terrestrial ecological systems based on linear fitting: a case study in Jiangsu, eastern China

**DOI:** 10.1038/s41598-019-53789-9

**Published:** 2019-11-20

**Authors:** Jianguo Li, Chenxin Zou, Qiang Li, Xinyue Xu, Yanqing Zhao, Wenhui Yang, Zhongqi Zhang, Lili Liu

**Affiliations:** 1 School of Geography, Geomatics, and Planning, Jiangsu Normal University, Xuzhou, Jiangsu 221116 China; 2 Department of Geography and School of Global Studies, University of Sussex, Falmer, Brighton UK

**Keywords:** Environmental impact, Sustainability

## Abstract

The terrestrial ecosystem productivity and foundation of regional ecosystem services have been significantly influenced by recent urbanization processes. This study assesses the changes in terrestrial ecosystem productivity in Jiangsu from the years of 2000 to 2015 in response to the urbanization. A linear model that incorporates the traditional equalization method is proposed to improve the estimation accuracy of net primary productivity (NPP) loss. Results revealed that the land area of urban construction expanded rapidly during the research period to encompass an area of 8672.8 km^2^. The rate of expansion was highest during 2005–2010. Additionally, the expansion rate of urban construction land was considerably higher in southern Jiangsu compared to the northern areas. The NPP exhibited a rising tendency from the year of 2000 to 2015, and varied from 33.30 to 40.23 Tg C/y. It was higher in the central parts, which include the cities of Yancheng and Nantong. The increase in urban construction land has resulted in a significant reduction in the terrestrial ecosystem productivity, i.e. a cumulative NPP loss of 2.55–2.88 Tg C during the research period. The NPP losses due to the conversion from cropland to constrction land were the highest, followed by the wetland. The work in this paper indicates that the rate of future productivity losses in terrestrial ecosystem in northern Jiangsu would be faster than the southern areas.

## Introduction

The productivity of terrestrial ecosystems is critical for regional carbon fixation and food supply capabilities^1–3^. Increasingly intensive and extensive human activities are significantly affecting terrestrial ecosystems. The impacts of anthropogenic activities on terrestrial ecosystems productivity and their future trends have been the primary subject of several studies^4–6^. In recent years, urbanization has been accelerating globally, especially in developing countries^7^. There is a profound impact on terrestrial ecosystems in the urbanization tends. Its processes, rates, and intensities are greatly affecting ecosystem’s structures, functions, consequents, and productivity. During the urbanization, large areas of wetland, cropland, forest, and grassland are converted rapidly into poorly permeable surfaces of cement and asphalt, which changes the regional environment and increases the risk of organic matter decomposition and release, further reducing ecosystem productivity^8–10^.

Net primary productivity (NPP) is a comprehensive indicator of regional productivity and is generally monitored using remote sensing techniques. In particular, following the launch of MODIS^11,12^, regional NPP can be estimated quickly and effectively using models such as the Carnegie-Ames-Stanford Approach (CASA), BEFS, C-FIX, and Biome-BGC as well as MODIS products, as demonstrated by multiple studies^11,13–15^. Urbanization processes can be depicted accurately using data from Landsat, CBERS, Huanjing, and other commercial satellites. Studies have shown that recent rapid urbanization in China has accounted for more than 60% of the total cropland yield loss due to land use and cover change (LUCC). The productivity of new cropland can compensate for the losses attributable to urbanization^16^. Several studies have been conducted in the regions and major metropolitan areas undergoing rapid urbanization in China^5,17–21^. Specifically, many studies have employed transition matrices coupled with average values of several years to investigate NPP variation in relation to the urbanization. However, the averaged values can lead to significant errors in characterizing urbanization processes^6,17,22^ as an urban expansion is a dynamic process. Thus, averaged urbanization measurements cannot objectively reflect NPP losses that have occurred during the corresponding years. Therefore, this study introduces a linear regression method to reduce estimation errors and increase estimation accuracy.

Jiangsu is an important developed province in eastern China where national strategies, such as the Coastal Tidal Flat Development, Yangtze River Economic Zone, Yangtze River Delta Metropolitan Region, and Huaihe Eco-Economic Corridor, have been implemented. Therefore, Jiangsu has become a core zone of rapid urbanization^23^. This region is an essential area for food production, therefore the rapid urbanization has had profound effect on the productivity of its terrestrial ecosystems. This study was conducted in Jiangsu and had two primary objectives: (1) to develop an effective method for estimating the NPP loss ascribed to the urbanization; (2) to improve the assessment accuracy of the relationship between the urban expansion and NPP loss.

## Results

### Urban construction land expansion in Jiangsu

**Figure 1:**
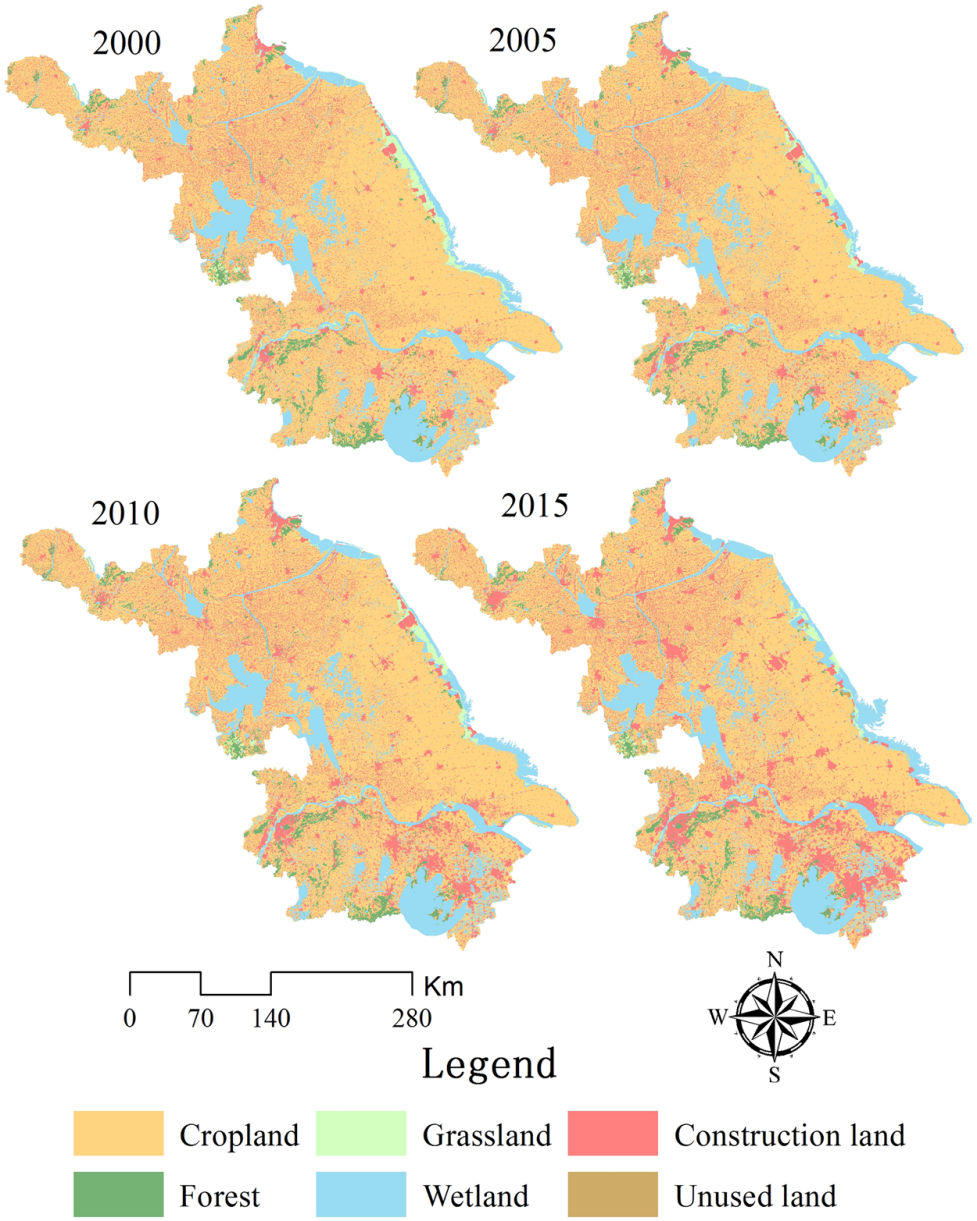
Land use change/cover map of Jiangsu Province (2000–2015). Map created using ArcMap 10.2 (ESRI, USA, https://www.esri.com/en-us/home).

The results showed that the magnitude and rate of urban expansion in Jiangsu differed considerably during the different time intervals (Table 1 and Fig. 1). Between 2000 and 2015, Jiangsu recorded slow expansion of urban construction land area (cumulative expansion: 8672.8 km^2^), but with relatively large fluctuations. The expansion magnitude and rate peaked in 2005–2010, when cumulative expansion reached 5909.53 km^2^ (average annual increase of approximately 1181 km^2^). The time interval 2000–2005 ranked second among the three intervals, with an expansion magnitude of 1471.85 km^2^ and rate of 294.37 km^2^/y. The expansion magnitude and rate were lowest in 2010–2015 at 1291.42 km^2^ and 258.2 km^2^/y, respectively. However, the rate of urban construction land expansion was 250 km^2^/y or more during the 15-year study period, which was relatively fast compared to other parts of eastern China. In Jiangsu, construction land expansion primarily involved the conversion of cropland which accounted for 92% of the total land use conversion. Wetland conversion, including lakes, rivers, tidal flats, and salt marshes ranked second; nearly 361.71 km^2^ of wetland were converted to urban construction land. In total, 130.33 and 112.27 km^2^ of forest and grassland, respectively, were converted and their rates of conversion were highest in 2005–2010. Unused land conversion to urban construction land was the smallest, accounting for only 15.43 km^2^. However, a gradual increase was observed over the study period; the converted area increased from 0 km^2^ in 2000–2005 to 13.77 km^2^ in 2010–2015.

**Table 1:** Scale and rate of urban construction land expansion in Jiangsu Province during different intervals (Units: km^2^ and km^2^/y, respectively).

	2000–2005 (km^2^)	Average annual (km^2^/y)	2005–2010 (km^2^)	Average annual (km^2^/y)	2010–2015 (km^2^)	Average annual (km^2^/y)	Total (km^2^)
C → B	1460.61	292.12	5443.48	1088.70	1148.95	229.79	8053.04
F → B	1.73	0.35	111.93	22.39	16.67	3.33	130.33
G → B	0.57	0.11	103.49	20.70	8.21	1.64	112.27
W → B	8.93	1.79	248.97	49.79	103.81	20.76	361.71
U → B	0.00	0.00	1.66	0.33	13.77	2.75	15.43
Total	1471.85	294.37	5909.53	1181.00	1291.42	258.20	8672.80

Note: B, C, F, G, W and U denote construction land, cropland, forest, grassland, wetland and unused land, respectively.

### NPP variations in jiangsu province

Table 2 shows a comparison of NPP values in the year 2000 for different plant functional types. Most of our derived NPPs is similar to those observed^24^, except for the farmland NPP. Our CASA model has underestimated the farmland NPP as compared to observation^24^. This is because in the CASA model, factors involving agricultural practices (e.g., fertilization, agricultural management, irrigation, etc.) have been excluded. In fact, these factors play an important role in promoting agricultural yield^25^. Compared to other studies, our derived NPPs for evergreen broad-leaf forest, deciduous broad-leaf forest and deciduous needle-leaf forest are very similar to the results of Ni^26^ and Liu^27^. However, our derived NPPs are significantly different from the result of Piao, *et al*.^2^. We speculate that this is mainly due to the difference in the maximum light use efficiency and other constraint factors, such as temperature and soil water content in our improved CASA model.

**Table 2:** Comparison of simulated NPP using the our CASA model with other studies (unit: gC/m^2^/y).

Vegetation type	Simulated	Observed	Piao, *et al*.^47^	Liu^27^	Ni^26^
	NPP	NPP			
Evergreen broad-leaf forest	996.1 ± 532.34	1016.5	525	945	945
Deciduous broad-leaf forest	712.8 ± 470.57	671.8	304	928	548
Deciduous needle-leaf forest	507.3 ± 356.81	490	432	585	460
Evergreen needle-leaf forest	485.1 ± 390.84	395.5	354	587	439
Farmland	742.3 ± 358.64	532.9	216	752	—
Plain grassland	289.8 ± 245.69	230.6	—	271	—

Mean ± SD.

**Figure 2:**
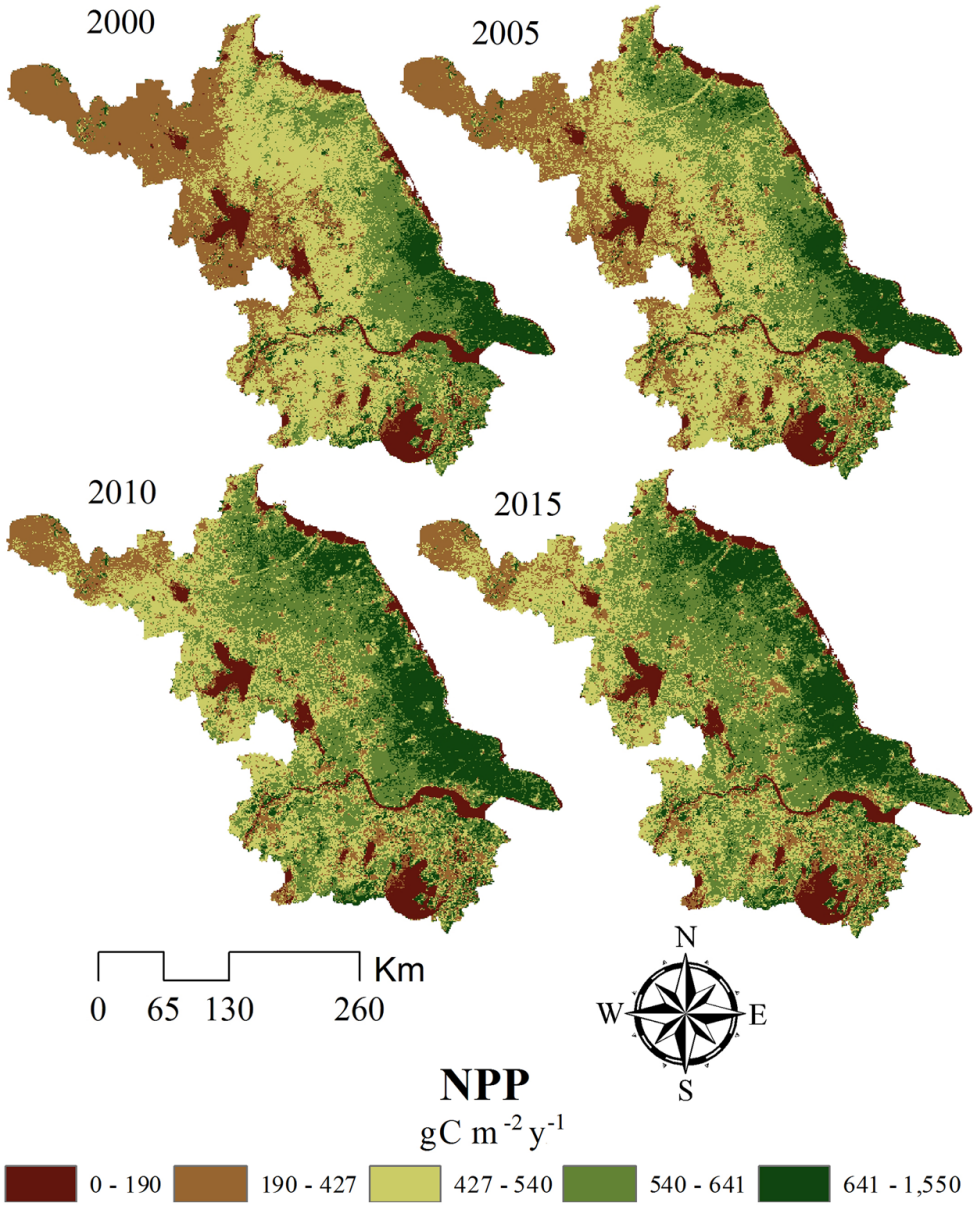
NPP of Jiangsu Province  in different years (2000–2015). Map created using ArcMap 10.2 (ESRI, USA, https://www.esri.com/en-us/home).

A slow increase in the NPP was detected in Jiangsu during 2000–2015 (Fig. 2). In general, NPP in the west was lower than the east, and NPP in the north and south was lower than the central areas. Relatively low NPP was recorded in Xuzhou, Huai’an, and to the south of the Yangtze River. Areas of high NPP were located in central Jiangsu, especially around the coastal cities of Yancheng and Nantong.

### Effect of urban construction land expansion on NPP

Equations 6–8 were used to calculate NPP losses during the three time intervals following the conversion of different land use areas to construction land (Table 3). The remote sensing images only recorded the initial and final NPP for the specified time intervals; therefore, the annual NPP change of different years were added to identify the total NPP change during the study period. The study assumed that urban construction land expansion followed a linear increase and adopted linear fitting equations to assess and sum the NPP of each year during the different time intervals (Fig. 2 and Table 3). In general, during 2000–2015, the NPP loss due to urban expansion initially decreased and then increased. The NPP losses in 2000–2005, 2005–2010, and 2010–2015 were 0.4, 0.38, and 1.77 Tg C, respectively. Thus, the highest losses occurred in 2010–2015 and the cumulative 15-year loss was 2.55 Tg C. The loss of cropland was the highest and related cumulative NPP loss was 1.63 Tg C, accounting for 64% of the total loss. During the three time intervals studied, Jiangsu experienced the highest loss in 2010–2015 (up to 1.77 Tg C); however, in 2005–2010, the city recorded the smallest loss. This small NPP loss was not in agreement with the considerable loss of cropland during this period. This was attributed to the concentration of construction land expansion in low-NPP regions to the south of the Yangtze River during this period. Cropland in this region is comparatively fragmented and scattered due to networks of lakes, rivers, and hills in southern Jiangsu. Further, the mixed-pixel problem is relatively serious in applications using remote sensing images with 1 km spatial resolution, which hindered the assessment of actual NPP losses during the study period^28^.

**Table 3:** Total NPP loss of Jiangsu Province caused by urban construction land expansion during different time intervals (Unit: Tg C).

	2000–2005 NPP loss (L_i_)	2005–2010 NPP loss (L_i_)	2010–2015 NPP loss (L_i_)	Cumulative NPP loss (L_i_)
C → B NPP loss	−0.12075	−0.01348	−0.35908	−1.63
Linear stretch	Y = −0.02415×	Y = 0.0268x − 0.14756	Y = −0.0864x + 0.07292	
F → B NPP loss	−0.00814	−0.002436	−0.0134	−0.091
Linear stretch	Y = −0.001628×	Y = 0.0014x − 0.0096	Y = −0.002741x + 0.0003	
G → B NPP loss	−0.00112	−0.000223	−0.00143	−0.011
Linear stretch	Y = −0.000224×	Y = 0.0002x − 0.0013	Y = −0.0003x + 0.00007	
W → B NPP loss	−0.00248	−0.003123	−0.31275	−0.81
Linear stretch	Y = −0.000496×	Y = −0.0002x − 0.0023	Y = −0.0774x + 0.07428	
U → B NPP loss	0	0.0000912	−0.00115	−0.0024
Linear stretch	0	Y = 0.00001824×	Y = −0.00031x + 0.0004	
Cumulative NPP loss (L_i_)	−0.40	−0.38	−1.77	−2.55

Note: B, C, F, G, W and U denote construction land, cropland, forest, grassland, wetland and unused land, respectively.

Additionally, relatively large NPP loss due to conversion of wetland (cumulative loss: 0.81 Tg C) was recorded during the study period; the losses were greatest during 2010–2015. Conversion of forest ranked third in the list. Although the exploited areas of forest and grassland were similar, their regional productivity losses differ considerably (by up to eight times), as forest has relatively higher NPP per unit area. The conversion of forest in the study area caused NPP losses of 0.09 Tg C during the 15-year period, whereas grassland conversion resulted in NPP losses of only 0.01 Tg C. The cumulative NPP loss due to the conversion of unused land to construction land was 0.0024 Tg C. In particular, the conversion during 2005–2010 caused an NPP increase of 0.00027 Tg C, whereas the conversion in 2010–2015 led to a loss of 0.0027 Tg C. This was mostly because green spaces were required in urban areas and additional vegetation was introduced at the initial conversion stage which increased the NPP of the region^29^. However, this increase was anthropogenic, and intended to cater to the green space requirement of construction land.

### Regional differences in NPP variations

The cumulative NPP increase for each city was obtained by calculating the differences in NPP between two consecutive years and adding them for the entire period of study (2000–2015), using Eq. (9) (Table 4). Although urbanization in Jiangsu reduced NPP of different land types considerably (−2.5Tg C), the cumulative NPP for the entire region showed an increase (0.2 Tg C). The primary reason was that regions which recorded NPP losses due to urbanization were located mostly near cities and were relatively small in area. Regions of high NPP such as forest, grassland, and cropland occupied larger areas and recorded an overall NPP increase. Thus, they effectively compensated the regional NPP losses and even contributed to an increase in the regional total. Yancheng and Yangzhou in northern Jiangsu demonstrated the highest increases in NPP of 0.58 and 0.35 Tg C, respectively. However, significant reduction in regional productivity was also observed in northern Jiangsu. The cumulative losses in Lianyungang and Suqian were 0.53 and 0.36 Tg C, respectively. Except for Xuzhou, all cities indicated total cumulative NPP increase higher than cumulative NPP losses due to urban construction land expansion. This reflects that NPP increase contributed by other land use types (forest, grassland, and cropland) largely compensated for the regional total losses caused by the cities. Xuzhou was an exception as it is the only coal-based city in Jiangsu; the rapid NPP reduction was largely due to large-scale coal mining during the past 30 years which has led to severe vegetation degradation and loss of cropland. This region is characterized by large amounts of stacked coal gangue, barren hills, and large-scale areas of subsidence. These factors compounded with rapid urbanization resulted in quick reduction in the terrestrial productivity in Xuzhou. Wuxi and Suzhou in southern Jiangsu also recorded notable losses in regional productivity of up to 0.35 and 0.29 Tg C, respectively.

**Table 4:** Regional total cumulative NPP change due to urban expansion (Unit: Tg C).

	City	2000–2005	2005–2010	2010–2015	Cumulative NPP loss	Total cumulative NPP change
Northern Jiangsu	Yangzhou	−0.02	−0.01	−0.02	−0.05	0.35
	Huai’an	−0.03	0.01	0.07	0.05	0.13
	Lianyungang	−0.01	−0.03	−0.49	−0.53	−0.25
	Nantong	−0.06	0.00	0.21	0.16	0.17
	Suqian	−0.03	−0.04	−0.29	−0.36	−0.34
	Taizhou	−0.11	−0.10	−0.14	−0.34	0.13
	Xuzhou	−0.01	−0.02	−0.22	−0.25	−0.94
	Yancheng	−0.02	−0.02	−0.20	−0.24	0.58
Southern Jiangsu	Changzhou	−0.02	−0.02	−0.06	−0.10	−0.06
	Nanjing	−0.04	−0.04	−0.07	−0.15	0.16
	Wuxi	−0.01	−0.04	−0.30	−0.35	−0.04
	Suzhou	−0.06	−0.05	−0.18	−0.29	0.15
	Zhenjiang	−0.01	0.00	−0.09	−0.10	0.16
	Cumulative NPP loss	−0.40	−0.38	−1.77	−2.55	0.20

## Discussion

Jiangsu Province is a typical alluvial plain with largely flat landform and lies mainly within the Yangtze River Basin and lower reaches of the Huaihe River Basin. Economic development has been rapid, with a GDP growth rate of 6.7% in 2018, exceeding the national average (6.6%). Construction land expansion in this region has been scarcely influenced by natural factors such as topography and natural hazards, and has been characterized by edge expansion and infill development^30,31^. Economic development in Jiangsu has been more efficient and at lower cost compared to other areas of China, resulting in faster urban construction land expansion compared to other cities^32^. The major drivers for this expansion include GDP increase, urban population growth, and fixed asset investments^33^. In particular, accelerated population growth has contributed heavily to the recent urbanization in Jiangsu (population urbanization). Rapid expansion of construction land has been inevitable to address growing employment and housing demands. Nevertheless, the expansion rate might slow in the future due to of rising environmental problems (e.g., air and water pollution and solid waste disposal) and social issues (e.g., unemployed farmers and social security costs). To address these problems, the government has implemented numerous land control measures such as the “Land Conservation and Intensive Land Use Promotion Program in Jiangsu Province” (also referred to as the “Double Up” plan, http://www.mlr.gov.cn/zwgk/zytz/201409/t20140926_1331065.htm). Its objective is to enhance the GDP of construction land by 50% and hence, improve development efficiency and quality, and reduce risk of inefficient development. Meanwhile, the introduction of policies such as the “Land Conservation and Intensive Land Use Promotion Program” have limited the overall increase of construction land expansion.

During the 15-year study period, construction land in Jiangsu expanded by 8672.8 km^2^ at an annual expansion rate of 578 km^2^. The source of this expansion has been mostly cropland (Table 5). The economy of southern Jiangsu is better developed than the north, and has resulted in rapid areal increase in urban construction land. On an average, each city has expanded by 56.88 km^2^/y, which is much faster than the average in the north during the same period (36.72 km^2^/y). Suzhou, Nanjing, and Nantong have grown most rapidly. Their individual cumulative areal expansion has been 1722.17, 842.2, and 794.74 km^2^, respectively, at annual rates of 114.8, 56.15, and 52.98 km^2^/y, respectively. Although the demand for urban construction land in southern Jiangsu is high, the rate of expansion may decrease in the future due to the aforementioned policies and the need to conserve regional land resources. However, urbanization in northern Jiangsu continues to accelerate as the constraints are less rigorous in this region; urban construction land expansion might continue. Therefore, the loss in productivity of terrestrial ecosystems in the north may exceed the south in the future.

**Table 5:** Sources of construction land converted in urban expansion in Jiangsu Province in 2000–2015 (Unit: km^2^).

City		C → B	F → B	G → B	W → B	U → B	Total
Northern Jiangsu	Yangzhou	430.27	1.33	2.67	3.94	0.00	438.22
	Huai’an	447.06	11.19	0.53	10.48	0.00	469.27
	Lianyungang	300.04	10.03	5.49	21.60	0.00	337.15
	Nantong	713.96	0.65	29.67	50.46	0.00	794.74
	Suqian	466.62	3.64	0.46	7.52	0.00	478.24
	Taizhou	499.02	0.33	0.00	9.32	0.00	508.67
	Xuzhou	589.15	20.56	5.37	17.01	0.37	632.48
	Yancheng	709.32	0.70	27.87	9.78	0.15	747.81
Southern Jiangsu	Changzhou	510.29	2.38	0.08	11.45	0.00	524.19
	Nanjing	783.20	35.42	3.84	19.29	0.44	842.20
	Wuxi	726.42	17.42	0.77	20.67	0.00	765.29
	Suzhou	1632.14	14.12	2.30	72.70	0.90	1722.17
	Zhenjiang	380.81	19.62	2.20	9.76	0.00	412.38
Total		8188.29	137.40	81.25	263.99	1.86	8672.8

Note: B, C, F, G, W and U denote construction land, cropland, forest, grassland, wetland and unused land, respectively.

Scales of urban construction land expansion are different in different regions. They mostly follow linear, logarithmic, nonlinear, or even exponential functions^34,35^. The corresponding annual NPP variations are even more complicated. Thus, it is very difficult to assess actual NPP losses due to urbanization. The objective of this study was to improve the estimation accuracy based on the assumption that urban construction land expansion follows a linear relationship. The results (Table 6) indicate that the estimates using linear fitting are often lower than estimates based on averages. The cumulative error in NPP loss during the 15-year study period was approximately 65%, highlighting the amplified effects of averaging long-term observational data. This is largely because averaging ignores dynamic processes and uncertainties during urban construction land expansion processes. Different cities in different periods show clear peculiarities in their expansion patterns. Therefore, estimation models based on different expansion characteristics should be studied in the future. Integrating such models and other nonlinear factors, such as fuzzy mathematics, system dynamics, and artificial neural networks will contribute to improved estimation of NPP loss due to urban expansion. Additionally, this study was based on 1-km MODIS data products. The evolution of fragmented spatial elements was not detected or monitored well due to the relatively low spatial resolution of the data. If constrained linear decomposition or nonlinear decomposition methods are used to decompose pixels for low-resolution data before estimation, the research results will be more accurate. This technique can be employed to improve future studies^36^.

**Table 6:** Regional productivity losses based on linear fitting and averaging.

	2000–2005 (Tg C)	2005–2010 (Tg C)	2010–2015 (Tg C)	Cumulative Loss (Tg C)
Results based on linear fitting	−0.40	−0.38	−1.77	−2.55
Results based on averaging^19^	−0.66	−0.1	−3.44	−4.20

Note: the method of averaging was addressed in literature of Jiang, *et al*.^19^.

**Figure 3:**
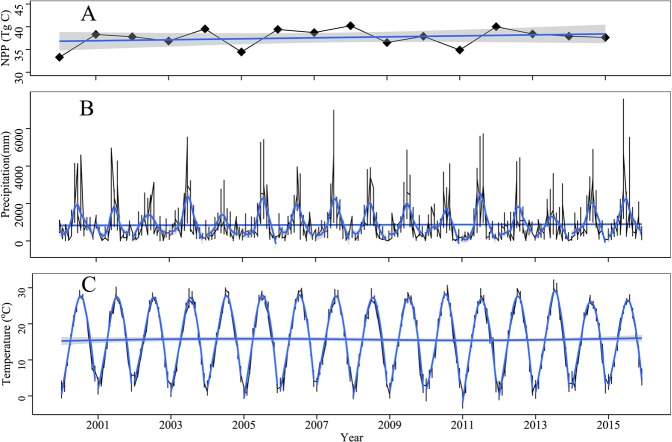
Total NPP (**A**), precipitation (**B**) and temperature (**C**) changes in Jiangsu Province during 2000–2015.

This study adopted the CASA model, improved by Zhu *et al*.^24^. However, the model accuracy depends on the vegetation type and water stress factors. Additionally, the study was restricted by the spatial resolution of the vegetation classification map (1:4 million); therefore, NPP estimation errors were unavoidable. Meteorological factors, especially temperature and precipitation, are primary factors that affect regional NPP variations^12,37–39^. As shown in Fig. 3, the total NPP in Jiangsu has increased slowly during recent years, whereas temperature has fluctuated little during the study period. Overall, temperature increased slowly and extreme precipitation became more frequent. Nitrogen deposition has been substantial in the study area as it experiences the highest nitrogen deposition in China^40–42^. Furthermore, new crop species and breeding technologies can compensate for the NPP losses due to cropland conversion and urban expansion^4^. Technological advancements such as fertilizers, innovations in agronomic measures and irrigation, and policies such as the “Ecological Red Line” and “High-Standard Prime Farmland” can promote regional NPP significantly and thereby reduce the risk of short-term NPP reduction due to urban construction land expansion. These factors may have contributed to the slow NPP increase in Jiangsu during the study period. The total NPP grew from 33.3 Tg C/y in 2000 to 37.65 Tg C/y in 2015, representing an increase of 4.35 Tg C (13.06%) during the 15 years. Based on this rate, the potential NPP loss due to urban construction land expansion in the past 15 years could have reached 2.88 Tg C. Some studies have suggested that rapid urbanization will lead to short-term regional productivity loss, but urban ecosystems will gradually compensate for the loss within 70–100 years^43^. This suggestion is based on the finding that regional urban heat islands, along with increased CO_2_ concentrations and nitrogen deposition, will lead to greater individual biomasses in the urban ecosystems compared to the surroundings. Field measurements in Taizhou of Zhejiang Province have demonstrated that the biomass of urban trees is twice that of tress of the same species in the field^20^. Thus, reasonable increase in vegetation coverage in urban areas could effectively compensate for the productivity loss due to urbanization and enhance carbon fixation capability.

We developed an improved method to estimate the loss in NPP caused by the recent urbanization in Jiangsu. We noted that the increase in urban construction land resulted in significant reduction in terrestrial ecosystem productivity with a cumulative NPP loss of about 2.55 Tg C during 2000–2015. Our results suggest that land use change continues to affect NPP of various land use types, especially near cities. Further, the rate of future terrestrial ecosystem productivity losses in northern Jiangsu would be faster than the southern parts of the province.

## Methods

### Study area

**Figure 4:**
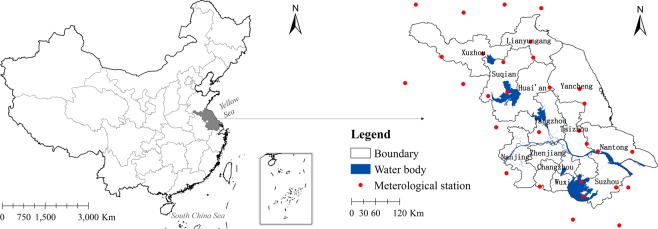
Map showing location of Jiangsu and meterological stations used in this study. Map created using ArcMap 10.2 (ESRI, USA, https://www.esri.com/en-us/home).

Jiangsu Province is located in eastern China (30°45–35°20′N, 116°18–121°57′E) and covers an area of 107.2 × 10^3^ km^2^. It is bordered by the Yellow Sea to the east, Shandong Province to the north, Henan and Anhui provinces to the west, and Zhejiang Province and Shanghai to the south. Its GDP was 9.26 × 10^3^ billion (CNY) in 2018, ranking second in China. Its urbanization level was 69.61%, approximately 10.0% higher than the national average. It has a temperate-subtropical transitional climate with four distinct seasons. The average temperature is 13–16 °C, which rises gradually from the northeast to the southwest. The average annual precipitation is around 1000 mm. The total cropland area is 4.60 × 10^6^ ha and the area of cropland per capita is 0.058 ha (Fig. 4).

### Data sources and CASA model

For NPP estimation, a CASA model was employed to estimate regional NPP and produced a temporal resolution of one month and spatial resolution of 1 km. Meteorological data (including monthly precipitation, average monthly temperature and monthly solar radiation) were obtained from the National Meteorological Information Centre of the China Meteorological Administration (http://data.cma.cn/). A total of 30 meteorological stations were imported and all cliamte datasets were interpolated dased on these meteorological stations (Fig. 4). LUCC data were acquired from the MCD12Q2 dataset and were in accordance with the IGBP land cover classification system and were used to simulate CASA-NPP. The 30-m land use data were acquired from the Yangtze River Delta Science Data Centre. LUCC data for 2015 were obtained by modifying LUCC polygons in the 2010 data according to Landsat 8 ETM^+^ images. All 30-m land use data were validated and had higher kappa coeffiecient (>0.7). 30-m land use data (Four land use images for 2000, 2005, 2010 and 2015 respectively) were used to obtain land use transition matrices and identify the extent and boundary of urbanization accurately. Compared with the MCD12Q2, 30-m land use data derived from landsat images generally had higher precision in spatial resolution which can provide detailed information on urbanlisation processes and further improve the accuracy of NPP loss estimation.

In this study, NPP was estimated using the improved CASA model established by Zhu *et al*.^24,44^, as described by the equation below:

1 $$NPP(s)=\sum APAR(x,t)\times \varepsilon (x,t),$$

2 $$APAR(x,t)=SOL(x,t)\times FPAR(x,t)\times 0.5,$$

3 $$FPAR(x,t)=1.08\times I(x,t)-0.08,$$

4 $$\varepsilon (x,t)={\varepsilon }_{{\rm{\max }}\_NPP}\times {T}_{\varepsilon }(x,t)\times {W}_{\varepsilon }(x,t),$$

5 $${W}_{\varepsilon }(x,t)=0.5+0.5\times E(x,t)/{E}_{{\rm{p}}}(x,t),$$

where *NPP*(*x*) is the NPP of pixel *x* (g C m^−2^ y^−1^), *APAR*(*x*, *t*) is the photosynthetically active radiation in month *t* in pixel *x* (g C/m^2^/month), and *ε*(*x*, *t*) is active light use efficiency in month *t* in pixel *x* (g C/MJ). *SOL*(*x*, *t*) is the total solar radiation in month *t* in pixel *x* (MJ/m^2^/month). *FPAR*(*x*, *t*) is the fraction of photosynthetically active radiation in month *t* in pixel *x*, representing the percentage of photosynthetically active radiation absorbed by plant. In this study, *FPAR* has been estimated based on the linear relationship between FPAR the NDVI (see Eq. 3)^24,45^. *I*(*x*, *t*) is the NDVI in month *t* in pixel *x*. Notably, bare soil gives an NDVI of 0.05 and but the NDVI in a closed, infinitely thick, green vegetation canopy was assigned as 0.9^45^. A factor of 0.5 is used to represent the proportion of active solar radiation to total solar radiation. ${\varepsilon }_{\max \_NPP}$ is a biome-specified variable, representing the maximum ability of a particular biome to convert absorbed radiation into dry matter. *T*_*ε*_(*x*, *t*) is temperature restriction factor and *W*_*ε*_(*x*, *t*) is water restriction factor. *E*(*x*, *t*) and *E*_*p*_(*x*, *t*) are actual and potential evapotranspiration in month *t* in pixel *x* respectively (mm/month). In this study, potential evapotranspiration is estimated using Penman-Montcith method^46^.

### Linear fitting for evaluating NPP loss

Spatial superimposition was used to identify areas of land types converted to urban construction land. Firstly, land use data of different years were superimposed to obtain polygons identifying areas that had changed into construction land during that period. Next, the total NPP difference of the polygons was calculated as:

6 $${L}_{{\rm{i}}}=N{{\rm{i}}}_{{\rm{t}}}-{{\rm{Ni}}}_{t-1},$$

Where, *L*_i_ denotes the loss in productivity of terrestrial ecosystems (i.e., NPP(t) here), and *N*i_t_ and *N*i_t-1_ represent the total NPP of polygons where the i-th land use type has been converted to construction land at time *t* and the total NPP of the corresponding polygons of the i-th land use type at time *t-1*, respectively.

**Figure 5:**
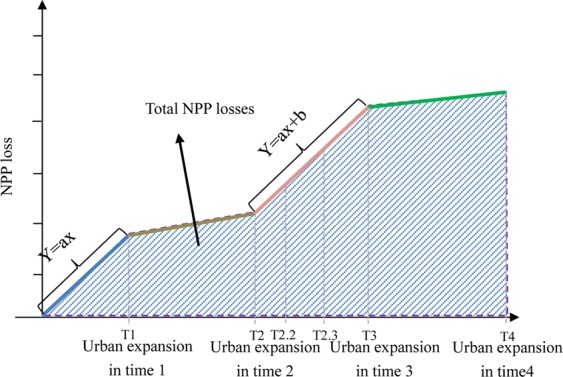
Linear fitting to assess NPP losses.

Three time intervals were considered in this study: 2000–2005, 2005–2010, and 2010–2015. Linear regression was employed to simulate the relationship between urban construction land expansion and NPP loss for each time interval (Fig. 5). The regression equations used are as follows:

7 $${\rm{Y}}=ax,$$

8 $${\rm{Y}}=ax+b,$$

where, *Y* represents the cumulative NPP loss for a specific land use type during a specific time interval (Tg C), *a* and *b* are linear regression parameters, and *x* is the cumulative urban construction land expansion during the time interval. Equation (7) was used for the initial interval (2000–2005), while Eq. (8) was used for the two subsequent time intervals.

### Cumulative NPP change

Cumulative NPP change was calculated using the equation below:

9 $$NP{P}_{tc}=\mathop{\sum }\limits_{i=2000}^{2015}NP{P}_{t}-NP{P}_{t-1}$$

where, *NPP*_*tc*_ represents the total cumulative NPP change during the time interval (2000–2015) (Tg C), *NPP*_t_ and *NPP*_t-1_ represent the total NPP of polygons at time *t* and total NPP of the corresponding polygons at time *t-1*, respectively.

### Software

In this study, all spatial analyses were conducted using ArcGIS 10.2 (ESRI Inc., USA) and statistical analyses (including liner fitting and descriptive statistics) were performed using R software.
